# 

**Published:** 1919-05-17

**Authors:** 


### Queen Charlotte's Hospital.

At the annual Court of Governors it was reported that the assistance of the hospital had been continued to soldiers' and sailors' wives, 291 having been received into the institution and 839 attended at their homes during the past year. The total during the war was nearly 5,000, and included a considerable number of wives of men in the overseas forces, as well as Belgian and other refugees. The Local Government Board had recognised the work done in the Ante-Natal Department and the Infant Consultation Centre, as well as in the District Midwifery Department, by making grants in aid of the expenses. The number of in-patients last year was 1,649, as compared with 1,947 in 1917. Special efforts had been made to obtain increased support, with the result that donations had increased from £2,644 to £4,222, the total income being £12,970, or £156 less than the expenditure. The total deficiency of the income and expenditure accounts accumulated during the war amounted to about £10,000.

### The College of Nursing (Limited by Guarantee).

VACANCIES ON COLLEGE COUNCIL.

Miss Bremner's Nomination.

Miss Geraldine Bremner, a member of the Nurses' Co-operation, and nurse representative on its Committee of Management, has been nominated by the London Centre of the College of Nursing as a candidate for election to the Council to represent nurses. She would like to thank all those who have kindly promised to support her by their votes and to assure them that in the event of being elected a member of the Council she will do her utmost to advance the interests of all nurses.

LINCOLN CENTRE.

(Hon. Secretary, Miss Sheppard, County Hospital, Lincoln.)

A meeting was held on May 3 at the County Hospital, Miss Sheppard, R.R.C., in the chair. It was proposed by Miss Wheeley and seconded by Miss Hobday that a resolution be forwarded to all local members of Parliament strongly opposing the Bill for State Registration, in its present form, now before the House of Commons. Notice was given of a lecture by C. J. Coleman, Esq., M.A., M.D., D.Ph., on "The Nursing of Venereal Diseases," on May 5. This lecture was very well attended. The next lecture will be given by Lieut.-Colonel W. H. Brook, R.A.M.C.T., on May 19. Subject: "The Rôle of Psychology in Nursing." The annual meeting of the Centre will be held on May 24, at 2.30 p.m., in the County Hospital, by kind permission of the Board of Management.

BRIGHTON AND HOVE CENTRE.

(Hon. Secretary, Miss A. M. Latham, Royal Sussex County Hospital, Brighton.)

This Centre is progressing admirably. Its membership is steadily increasing, but the Hon. Secretary will be pleased for as many more College nurses to join as possible. The Centre has obtained a club-room at No. 2 Old Steine, Brighton, which may now be used free of charge by members of this or any other Centre. Light refreshments are provided at a moderate price. It is hoped that, later, the Club may become residential, and with this object in view the Executive Committee have decided to hold a bazaar in December, and will be pleased of assistance from any who care to help. The first of a series of lectures will be given at the Pioneer Club, 4 New Road, Brighton, on Thursday, May 22, at 7.30 p.m., on "Cerebro-Spinal Meningitis," by Colonel Hobhouse, R.A.M.C. The lecture will be free to all members of the Centre, but a fee of 1s. will be charged to each one not a member of the Centre. Light refreshments, free of charge, will be provided after the lecture.

### MATRONS' AND SISTERS' APPOINTMENTS.

Aut-Even Hospital, Kilkenny, Ireland.—Miss Desiree M. Layng has been appointed matron. She was trained at University College Hospital, where she was later acting sister of a military ward.

David Lewis Northern Hospital, Liverpool.—Miss Margaret Middlemiss, Miss Jeannette B. Hunter, and Miss Marjorie Lingwood Mansell have been appointed sisters of the men's surgical ward, the men's medical ward, and the pensioners' ward respectively, and Miss Hannah Griffith has been appointed holiday sister. Miss Middlemiss was trained at Bradford Royal Infirmary, later being sister there, has been sister under the Scottish Women's Hospitals, and the Royal Naval Nursing Service Reserve. Miss Hunter was trained at the Norfolk and Norwich Hospital, and has been staff nurse and sister at the West London Hospital, Hammersmith. Miss Mansell was trained at the West London Hospital, Hammersmith. Both Miss Hunter and Miss Mansell have been doing tuberculosis work under the Welsh National Memorial Association. Miss Griffith was trained at Chester Royal Infirmary, where she has taken sister's holiday duties, and has been staff nurse in the Q.A.I.M.N.S.R.

Devonshire Hospital, Buxton.—Miss M. G. Gilkes-Robinson has been appointed matron. She was trained at Brentford Infirmary, has been matron of the Royal Albert Hospital, Devonport, assistant matron of the Evington War Hospital, Leicester, matron of a General Hospital in East Africa, superintendent of the Electrical Baths, Buxton, and assistant matron at the Devonshire Hospital.

District Hospital, Shepton Mallet.—Miss Violet A. Townsend has been appointed matron. She was trained at the Bristol Royal Infirmary.

Essex County Hospital, Colchester.—Miss McGowan has been appointed sister of the children's surgical ward. She was trained at the County Hospital, York, where she has been sister of the children's ward; and she was a member of the Q.A.I.M.N.S.R.

Infectious Diseases Hospital, Llantwit Fardre, near Pontypridd.—Miss Edith Cutter has been appointed matron. She was trained at the Borough Isolation Hospital, Huddersfield, has been matron of the Joint Isolation Hospital, Conway, of the Isolation Hospital, Skipton, and has done military nursing under the French Red Cross.

Jessop Hospital for Women, Sheffield.—Miss Nellie Horton has been appointed night sister. She was trained at St. Mary's Hospital, Paddington; has been staff nurse and sister at the Birmingham and Midland Hospital for Women; has done sister's duties at the Maternity Hospital, Birmingham; and has also done military nursing. She holds the certificate of the Central Midwives Board, and is a member of the College of Nursing.

Kent and Canterbury Hospital, Canterbury.—Miss Smales has been appointed matron She was trained at King's College Hospital, London, where she was subsequently sister. Additional appointments held were those of assistant matron at Queen's Hospital for Children, and sister, assistant matron, and matron in the T.F.N.S. She served two years in Salonica, was mentioned in despatches March 1917, awarded R.R.C., First Class, June 1917, and the Serbian Order of St. Sava October 1917.

Macclesfield General Infirmary.—Miss Mabel Rowley has been appointed night sister. She was trained at the General Infirmary, Bolton, and at Monsall Fever Hospital, Manchester. She has been holiday charge sister at the Auxiliary Military Hospital, Flixton; school nurse to the Eccles Borough Education Committee; night sister at the City Fever Hospital, Coventry; and has done private nursing. Miss Rowley is a member of the College of Nursing.

Maternity and Children's Hospital, Ilkeston.—Miss A. Hopwood, A.R.R.C., has been appointed matron. She was trained at Crumpsall Infirmary, later being sister there, and has been matron of the Second Western General Hospital, Morton, Manchester.

Municipal Babies' Home, Derby.—Miss Jane Elizabeth Haighton has been appointed sister. She was trained at St. Bartholomew's Hospital, London, has been sister at the Grove Military Hospital, Tooting, and sister-in-charge at the V.A.D. Hospital, Creswell, Nottingham.

Royal Infirmary, Wigan.—Miss Louisa Murray has been appointed sister. She was trained at the Royal Alexandra Infirmary, Paisley, where she was later ward sister and acting night sister. She has also been doing Army nursing.

Royal Waterloo Hospital for Children and Women, Waterloo Road.—Miss A. C. Rastall, R.R.C., has been appointed matron. She was trained at the Sheffield Royal Hospital, and has been matron of the Cameron Hospital, West Hartlepool, the Grimsby and District Hospital, and of the Princess Alice Hospital, Eastbourne.

